# Directed evolution of SIRT6 for improved deacylation and glucose homeostasis maintenance

**DOI:** 10.1038/s41598-018-21887-9

**Published:** 2018-02-23

**Authors:** Or Gertman, Dotan Omer, Adi Hendler, Daniel Stein, Lior Onn, Yana Khukhin, Miguel Portillo, Raz Zarivach, Haim Y. Cohen, Debra Toiber, Amir Aharoni

**Affiliations:** 1 Department of Life Sciences, Ben-Gurion University of the Negev, Be’er Sheva, 84105 Israel; 2 The Mina & Everard Goodman Faculty of Life Sciences, Bar-Ilan University, Ramat-Gan, 5290002 Israel; 3 The National Institute for Biotechnology in the Negev, Ben-Gurion University of the Negev, Be’er Sheva, 84105 Israel; 4 Present Address: Smartzyme Innovation LTD, Ilan Ramon, Science Park-Ness Ziona, Ness Ziona, Israel

## Abstract

Mammalian SIRT6 is a well-studied histone deacetylase that was recently shown to exhibit high protein deacylation activity enabling the removal of long chain fatty acyl groups from proteins. SIRT6 was shown to play key roles in cellular homeostasis by regulating a variety of cellular processes including DNA repair and glucose metabolism. However, the link between SIRT6 enzymatic activities and its cellular functions is not clear. Here, we utilized a directed enzyme evolution approach to generate SIRT6 mutants with improved deacylation activity. We found that while two mutants show increased deacylation activity at high substrate concentration and improved glucose metabolism they exhibit no improvement and even abolished deacetylation activity on H3K9Ac and H3K56Ac in cells. Our results demonstrate the separation of function between SIRT6 catalytic activities and suggest that SIRT6 deacylation activity in cells is important for glucose metabolism and can be mediated by still unknown acylated cellular proteins.

## Introduction

The sirtuins enzymes belong to a family of NAD^+^- dependent deacetylases that target a variety of acetylated proteins in mammals to regulate their cellular activity^1^. The founding member of the sirtuin family is Sir2 that was discovered in *Saccharomyces cerevisiae* and was shown to enhance the yeast life span^2^. In mammals seven sirtuins exists, including SIRT1–7, playing key roles in diverse cellular functions including metabolism, stress resistance, genome stability and tumorigenesis^1,3–5^. Each mammalian sirtuin exhibits distinct cellular localization, catalytic activity and substrate specificity.

SIRT6 is a prominent mammalian sirtuin that attracted substantial attention in the last decade. SIRT6 was shown to be involved in a variety of cellular processes such as glucose homeostasis, DNA repair and longevity^6^. SIRT6 deficient mice exhibit acute hypoglycemia, decreased insulin levels and severe degenerative processes leading to death at ~1 month old^7^. In contrast, overexpression of SIRT6 in mice was shown to increase male mice life span by ~15% probably by affecting mice glucose homeostasis^8^. Enzymatic characterization of SIRT6 revealed three catalytic activities: deacetylation, fatty acid deacylation and ADP-ribosylation. The *in vitro* histone deacetylation activity of SIRT6 on peptide substrates is relatively weak and is estimated to be three orders of magnitude lower than SIRT1 deacetylation^9,10^. However, SIRT6 deacetylation activity on nucleosomes or in the presence of fatty acid activators was shown to be significantly enhanced^9,11^. In contrast, SIRT6 deacylation activity is estimated to be at least two orders of magnitude higher than its deacetylation due to increased affinity for the fatty acid moiety^10^.

Due to the diverse biological roles and the three enzymatic activities of SIRT6 it is difficult to attribute a specific SIRT6 enzymatic activity to SIRT6 biological function in cells. Recently, separation of function SIRT6 G60A mutant^12^ was generated that exhibits native SIRT6 level of deacylation activity but significantly reduced deacetylation activity^13^. This mutant was shown to increase the secretion of a variety of proteins from cells probably due to direct/indirect effects of fatty acid deacylation of SIRT6 substrates. However, the specific effect of SIRT6 deacylation activity on its physiological functions in cells is still poorly understood. In addition, it is unknown whether increase in SIRT6 deacylation activity will lead to improved cell physiology.

Here, we utilized a directed evolution approach for the generation of SIRT6 variants with enhanced deacylation activity. Previously, directed evolution proved to be highly efficient methodology for the generation of enzymes with improved catalytic efficiency, altered specificity and improved stability^14–16^. Directed evolution is based on two main steps; the generation of gene library containing mutations in the target gene followed by screening/selection for variants with improved catalytic efficiency or altered substrate specificity^17,18^. Application of directed evolution methodology for SIRT6 allowed us to generate two SIRT6 mutants with enhanced deacylation activity at high substrate concentration. Interestingly, one of these mutants exhibits WT level of histone deacetylation activity while the other, exhibits a dramatic reduction in histone deacetylation. Phenotypic analysis of these variants in cells revealed that both mutants exhibit a significant increase in glucose homeostasis activity relative to the WT SIRT6. These results suggest that in addition to SIRT6 H3K9 deacetylation activity, the repression of glucose metabolism in cells is mediated by acylated SIRT6 substrates. Our results suggest that a specific enhancement in SIRT6 deacylation activity by mutations and possibly small molecule activators can lead to beneficial physiological effects.

## Results

### Generation of SIRT6 ancestral library for directed evolution

In the past few years, focused gene libraries proved to be highly beneficial for directed enzyme evolution, particularly, when high-throughput screening for enzymatic activity is difficult to establish^19^. These libraries are generated by integrating different levels of information on the protein of interest including structural, functional and evolutionary information. Recently, we have extensively utilized back-to-consensus focused gene libraries based on sequence alignment information for the isolation of proteins with improvements in stability and catalytic activity or binding affinity^20–22^. Here, we generated SIRT6 ancestral library based on SIRT6 ancestral reconstructed sequence for the identification of mutations that took place during natural SIRT6 evolution. We substituted various positions in human SIRT6 (see below) based on amino acid identities that were predicted to have existed in ancestral SIRT6 sequences. Due to the relatively weak catalytic efficiency of SIRT6, we hypothesized that amino acid substitutions based on ancestral sequences of highly active sirtuins (e.g. SIRT1) will lead to the isolation of SIRT6 variants with improved catalytic efficiency. Previously ancestral libraries were generated for the directed evolution of various enzymes including polymerases^23^, paraoxonase1 and sulfotransferase1^24^, leading to the isolation of enzymes with high functional diversity and improved catalytic activities.

**Figure 1:**
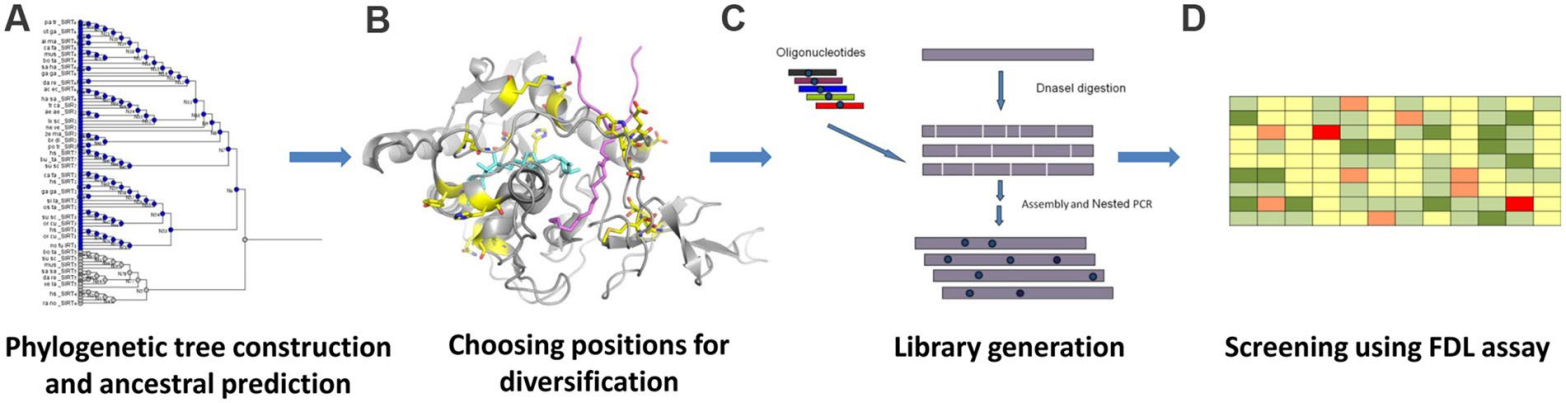
Scheme describing the directed evolution process for the generation of SIRT6 mutants with enhanced catalytic activity. The process includes: (**A**) The generation of phylogenetic tree and the prediction of ancestral SIRT6 sequences, (**B**) Selecting positions for diversification based on the distance from the catalytic H133 residue (see Supplementary Table S1 for the list of mutations), (**C**) Generation of ancestral library by spiking of nucleotides containing mutations such that each variant contains 3–5 mutations from the 27 possible substitutions selected, (**D**) Screening of the SIRT6 library in a 96-well plate format using the FDL assay with TNF-α myristoylated peptide.

To generate the SIRT6 ancestral library, multiple sequences of the deacetylase domain of the sirtuin family members were obtained. All sequences with identity lower than 45% or higher than 95% and truncated sequences were removed. A multiple sequence alignment (MSA) was generated based on 89 compatible SIRTs sequences that was manually improved based on the crystal structures of several SIRTs including SIRT6^10,25^. The MSA was then used to generate a phylogenetic tree of the SIRTs family to enable the prediction of the ancestral sequences at the different nodes (Fig. 1A). To construct SIRT6 gene library we chose the sequence of ancestral node 6 (N6) that exhibits 45% sequence identity with human SIRT6 with high conservation of the active site residues. The N6 node encompasses some of the present SIRT1, SIRT2 and SIRT3 sequences that were shown to exhibit the highest catalytic activity *in vitro* toward acetylated peptides (Supplementary Fig. S1). To narrow down the library size and maximize the possibility of improving SIRT6 catalytic activity, we selected 20 positions in SIRT6 for diversification located at a distance of up to 15 Å from the catalytic histidine residue (H133), based on the crystal structure of the wild type SIRT6 (PDB: 3ZG6) (Fig. 1B and Supplementary Fig. S2). SIRT6 gene library containing subsets of 27 substitutions (Supplementary Table S1) was generated by partial mutagenesis of the SIRT6 gene as previously described^21,26^. We sequenced five random SIRT6 variants from the resulting library and identified 3–6 ancestral mutations per gene (Fig. 1C).

### Screening of the SIRT6 ancestral library using Fluor de Lys (FDL) assay

To enable the screening of SIRT6 mutants in a 96-well plate format, we established a FDL screening assay for SIRT6 deacylation activity using crude *E. coli* lysates^27^. This assay enabled the robust detection of SIRT6 mediated deacylation of TNF-α K20myr peptide fused to the fluorescent probe 7-amino-4-methylcoumarin (AMC). However, using this assay, we were not able to monitor SIRT6 deacetylation of H3K9ac and H3K56ac FDL peptides. The lack of sensitivity for monitoring SIRT6 deacetylation activity in crude E. coli lysates is consistent with the previously observed very high K_M_ for these peptide substrates^10^. Using the FDL assay with TNF-α K20myr peptide, we screened ~1000 SIRT6 mutants in crude cell lysates (Fig. 1D). We identified 20 SIRT6 mutants exhibiting significant improvement in activity of at least 2 fold relative to the WT. These variants were re-screened in triplicates for verification and 7 successful mutants were purified by affinity chromatography on a small scale (Supplementary Fig. S3). Examining the activity of the purified mutants enabled us to normalize SIRT6 activity to the protein level and focus on two SIRT6 variants D1 and 6A4 that consistently demonstrated higher catalytic efficiency compared to the WT (Supplementary Fig. S3**)**.

### Examination of SIRT6 activity using HPLC kinetic assay and structural analysis

To further characterize the improvements in SIRT6 deacylation activity, the D1 and 6A4 mutants were overexpressed in *E. coli*, purified and characterized using HPLC activity assay. As a substrate, we used an unmodified 13 amino-acid peptide derived from the human TNF-α K20myr. Initial rates for SIRT6 activity were measured following SIRT6 incubation with various concentrations of TNF-α K20myr and the product was separated and quantified by the HPLC (Supplementary Fig. S4). To derive the k_cat_ and K_M_ parameters, the initial rate values were plotted against substrate concentrations and fitted to the Michaelis-Menten equation (Supplementary Fig. S5). We found that the D1 and 6A4 variants exhibit a significant increase in k_cat_ of 88% and 72%, respectively (Table 1). In addition, 6A4 exhibits an increase of ~3 fold in its K_M_ leading to reduced overall catalytic efficiency (k_cat_/K_M_) relative to the WT. The improvements in k_cat_ values of D1 and 6A4 are expected due to the relatively high concentration of TNF-α peptide of 50 μM used during the screening assay. To examine whether the improvements in SIRT6 deacylation activity is specific for TNF-α K20myr peptide, we examined the activity of D1 and 6A4 variants with H3K9myr peptide and compared it to SIRT6 WT activity (Supplementary Fig. S5). We found that D1 and 6A4 exhibit improved activity (increased k_cat_) also toward the H3K9myr indicating that improved deacylation activity is not significantly dependent on the peptide sequence (Table 1). Finally to examine whether the improvements in D1 and 6A4 deacylation activity is also accompanied by a lower K_M_ for the NAD^+^ cofactor, we examined the K_M_ for NAD^+^ under saturated TNF-α K20myr peptide concentrations. We found that D1 and 6A4 exhibit increased K_M_ for the NAD^+^, relative to the WT, indicating no improvement in NAD^+^ binding affinity (Supplementary Fig. S6–7).

**Table 1:** Catalytic parameters of SIRT6 variants with TNF-α K20myr and H3K9myr peptides.

SIRT6	Peptide	k_cat_ (sec^−1^)	% of WT	K_M_ (µM)	% of WT	k_cat_/K_M_ (sec^−1^*M^−1^)	% of WT
WT	TNFα myr	0.0033 ± 0.0002	100	8.1 ± 1.9	100	407	100
WT	H3K9 myr	0.0163 ± 0.002	100	14.6 ± 4.6	100	1116	100
D1	TNFα myr	0.0062 ± 0.0004	188	8.1 ± 1.5	100	765	188
D1	H3K9 myr	0.0273 ± 0.001	167	13.7 ± 2.3	93	2002	179
6A4	TNFα myr	0.0057 ± 0.0005	172	23.7 ± 4.7	292	240	59
6A4	H3K9 myr	0.0217 ± 0.001	133	18.23 ± 1.6	125	840	75

**Figure 2:**
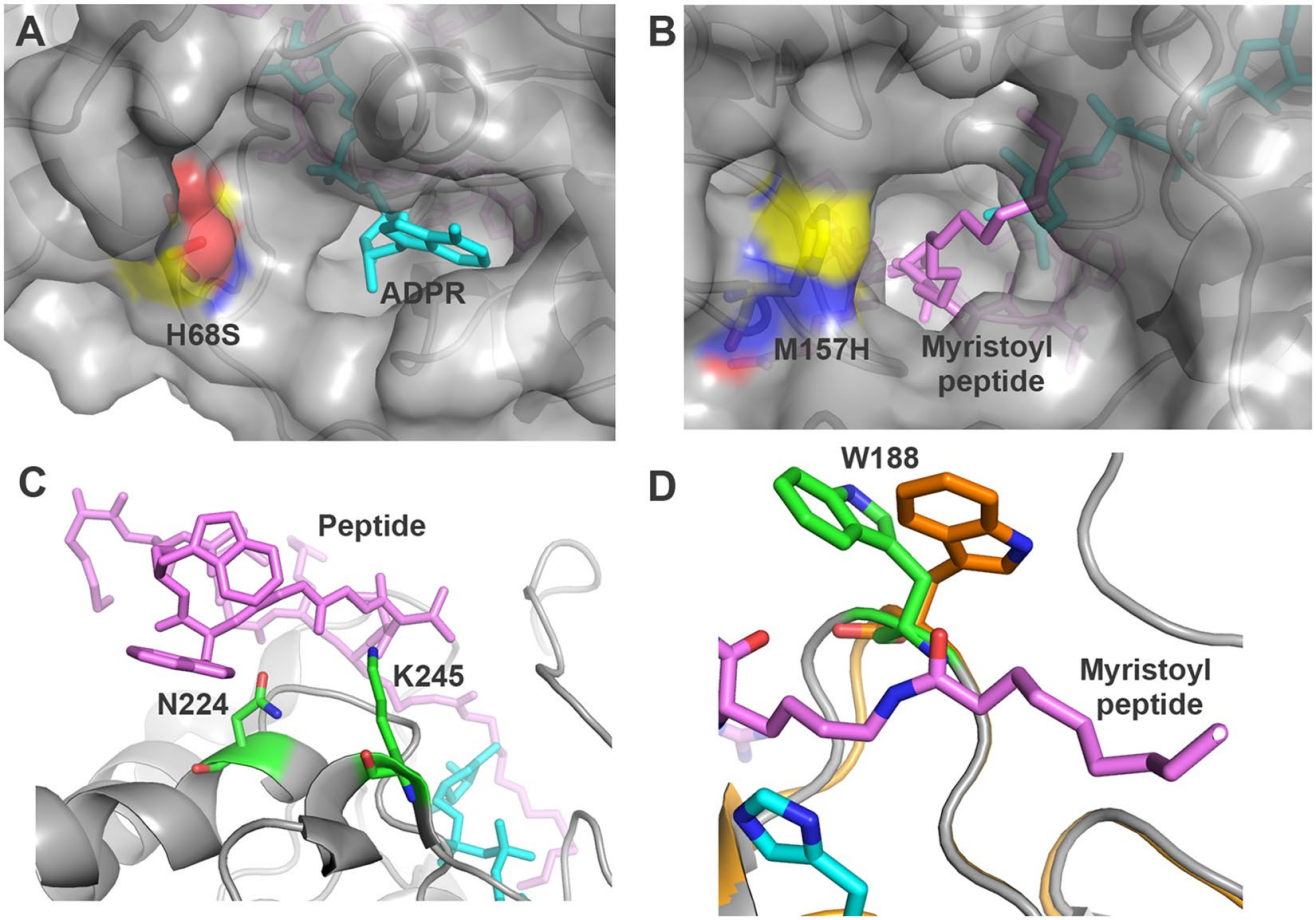
Structural analysis of residues that are mutated in the improved D1 and 6A4 SIRT6 variants. (**A**) Surface view of H68 region located in the vicinity of the NAD^+^ binding pocket. (**B**) Surface view of M157 region located in the vicinity of the myristoyl moiety of the H3K9myr peptide. The H68S and M157H mutations found in D1 and 6A4 variants were generated in silico. Both mutations can contribute to improved k_cat_ by better positioning of the peptide and NAD^+^ for catalysis. (**C**) Residues K245 and N224 are located in the vicinity of the peptide backbone and can contribute to the low K_M_ for the myrisoylated peptide. The K245P and N224S can eliminate interactions with the peptide in 6A4 leading to an increase in K_M_ for the TNF-α peptide. (**D**) Residue W188 undergoes a significant conformational change upon peptide binding. The movement of W188 was identified by superposition of SIRT6 structure with myristoylated peptide (PDB 3ZG6) and without a peptide (PDB 3K35). The W188F mutation found in D1 can lead to better positioning of the peptide with respect to the catalytic H133 (magenta).

Sequence analysis of the two mutants revealed that they share two common mutations, H68S and M157H. In addition, D1 contains the W188F mutation and 6A4 contains N224S and K245P mutations. Mapping these positions on the crystal structure of SIRT6 (PDB 3ZG6^10^) revealed the location of the mutations with respect to the peptide substrate, the acyl chain and the NAD^+^ cofactor binding pocket. We found that the two common mutations, H68S and M157H, are located in the vicinity of the NAD^+^ cofactor and the acyl chain of the TNF-α peptide, respectively (Fig. 2AB). These mutations can lead to increased k_cat_ of D1 and 6A4 due to improved positioning of the substrate for catalysis relative to the WT protein. The increase in K_M_ for 6A4 relative to the WT can stem from the N224S and K245P mutations that are located in the vicinity of the peptide backbone and can result from elimination of favorable interaction (Fig. 2C). Interestingly, we found that W188 residue that is mutated in D1 undergoes significant conformational change upon peptide binding^10,28^. This residue can enhance catalysis by shielding the active site from the water solution and its mutation to phenylalanine can provide better peptide positioning (Fig. 2D). However, future investigation must be performed to understand the structural changes due to SIRT6 mutations in D1 and 6A4 variants.

### Analysis of SIRT6 induced secretion of TNF-α from cells

**Figure 3:**
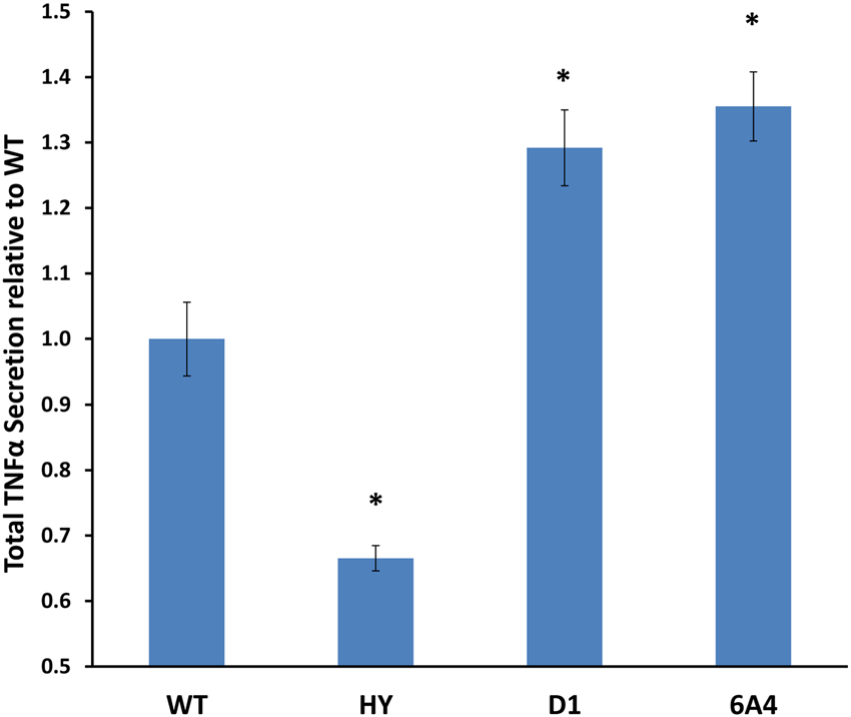
SIRT6 mutants lead to enhanced TNFα secretion to the medium. KO SIRT6 293T were co-transfected with human TNFα and SIRT6 variants. Secretion of TNFα to the medium was measured using ELISA and normalized to the expression of each SIRT6 variant using western blot analysis followed by Image J quantification of the level of each SIRT6 variants. The data presented is the average of three technical repeats of each experiment while the error bars represent the standard deviation from the average. Statistically significant differences of the different SIRT6 variants or the KO from the WT TNF-α secretion levels are labeled with black star (p < 0.001).

Previous studies have shown that SIRT6 is involved in the secretion of the TNF-α cytokine to the media through deacylation of K20myr^10^. To test whether the improvements in D1 and 6A4 deacylation activity measured *in vitro* are correlated with improvements in SIRT6 deacylation activity in cells, we examined the ability of these mutants to increase TNF-α secretion from cells. To reduce any background activity from endogenous SIRT6 in cells, we generated a SIRT6 knock-out (KO) 293T cell line using the CRISPR/CAS9 technology^29,30^. *SIRT6* KO in these cells was verified by the absence of SIRT6 protein leading to a significant increase in H3K56 acetylation level (Supplementary Fig. S8). To examine the level of TNF-α secretion induced by the SIRT6 improved mutants, we co-transfected the *SIRT6* KO cells with plasmids encoding for the human TNF-α and the different SIRT6 variants. Using TNF-α ELISA and SIRT6 western blot analysis, we normalized the levels of TNF-α secretion relative to SIRT6 expression. We found that D1 and 6A4 SIRT6 mutants exhibit ~1.3 fold increase in TNF-α secretion from those cells expressing WT SIRT6 that is in good correlation with the *in vitro* activity of these mutants toward TNF-α K20myr peptide (Fig. 3).

### Examination of SIRT6 activity with H3K9ac and H3K56ac in cells

**Figure 4:**
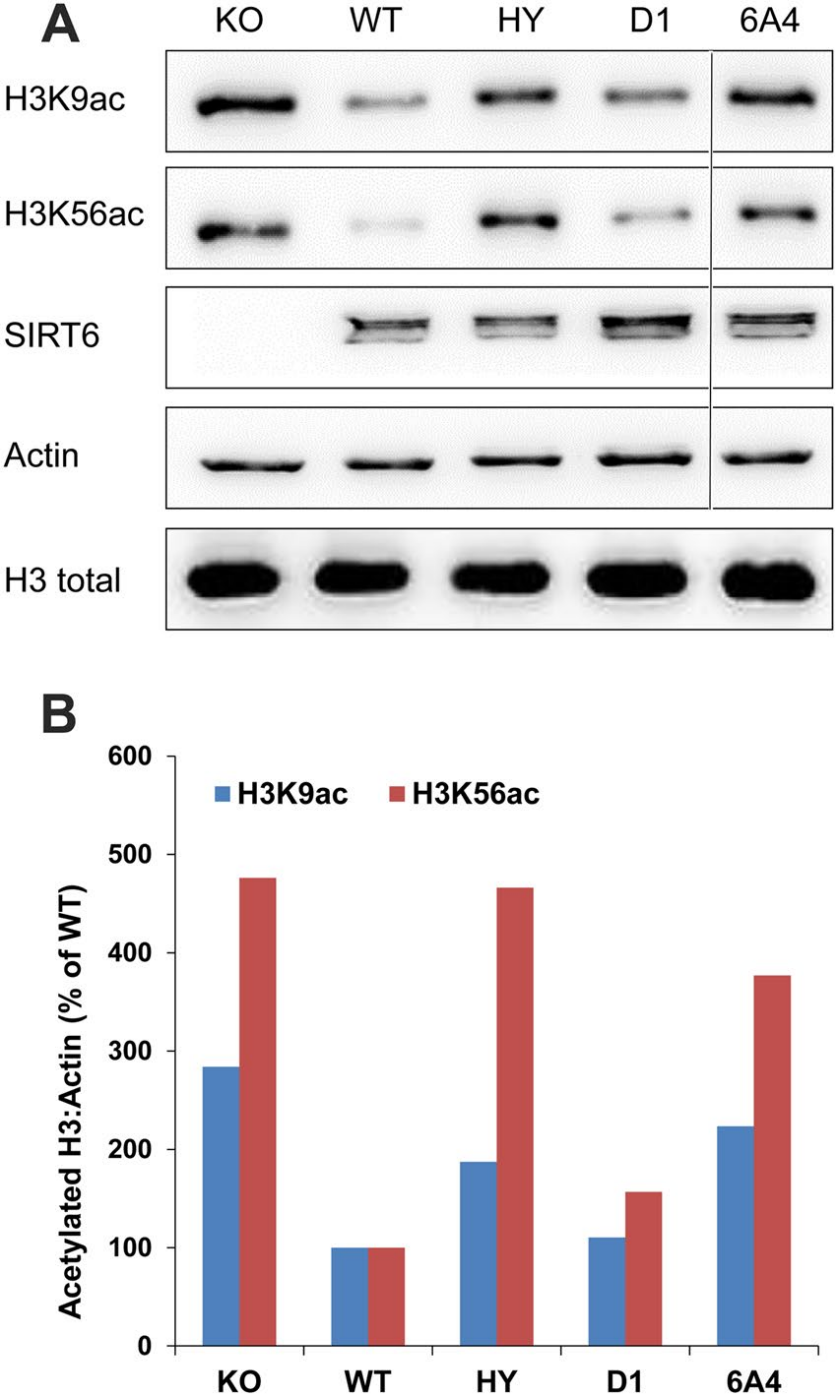
SIRT6 deacetylation of H3K9Ac and H3K56ac in MEFs. SIRT6 deacetylation activity was measured using western blot analysis with specific antibodies. Analysis was performed on crude cell lysates prepared from equal amount of KO MEFs cells that stably express the different SIRT6 variants, including WT, D1, 6A4 and the non-catalytic H133Y (HY). (**A**) Western blot analysis, the line highlights the deletion of non-relevant mutant’s analysis from the gel (original blots are shown in Supplementary Fig. S9) (**B**) Quantification of the western blot by image J to assess the activities of the D1 and 6A4 mutants relative to the WT and HY mutant. The western blot analysis is a representative gel from three independent repeats.

Previously, SIRT6 was shown to efficiently remove acetyl group from H3K9ac and H3K56ac in cells. SIRT6 deacetylation of H3K9ac gene promoters was shown to alter gene expression affecting cellular phenotype^31^. To examine whether D1 and 6A4 mutants with improved deacylation activity also exhibit improved deacetylation activity with H3K9ac and H3K56ac in cells, we examined the later activity in mouse embryonic fibroblasts (MEFs) cells. To eliminate any background SIRT6 activity in this cell line, we first generated and verified cell line containing *SIRT6* KO (Fig. 4A, left lane). These cells were then infected with retrovirus containing the WT, D1, 6A4 and the inactive H133Y mutant to generate stable MEF cell lines that constitutively express the different SIRT6 variants. To examine the level of H3K9 and H3K56 acetylation, equal amount of cells from the different cell lines were lysed and subjected to western blot analysis. Interestingly, we did not detect any improvement in the deacetylation activity of these mutants relative to the WT in this assay. Moreover, in the case of the 6A4 mutant we observed a significant decrease in the deacetylation with H3K9ac and H3K56ac that is similar to the H133Y mutants (Fig. 4 and Supplementary Fig. S9). To further examine D1 and 6A4 deacetylation activity, we have monitored their deacetylation activity on histone octamers isolated from HEK293 cells at different time points. In accordance with the activity of the mutants in MEFs, we found that D1 shows a minor decrease in the deacetylation of H3K56ac while 6A4 exhibit no detectable deacetylation activity (Supplementary Figs S10–S11). Overall, these results indicate that the mutations in D1 and 6A4 led to a specific improvement in SIRT6 deacylation activity with none or negative effect on SIRT6 deacetylation activity, respectively.

### D1 and 6A4 repress glucose metabolism in MEFs

**Figure 5:**
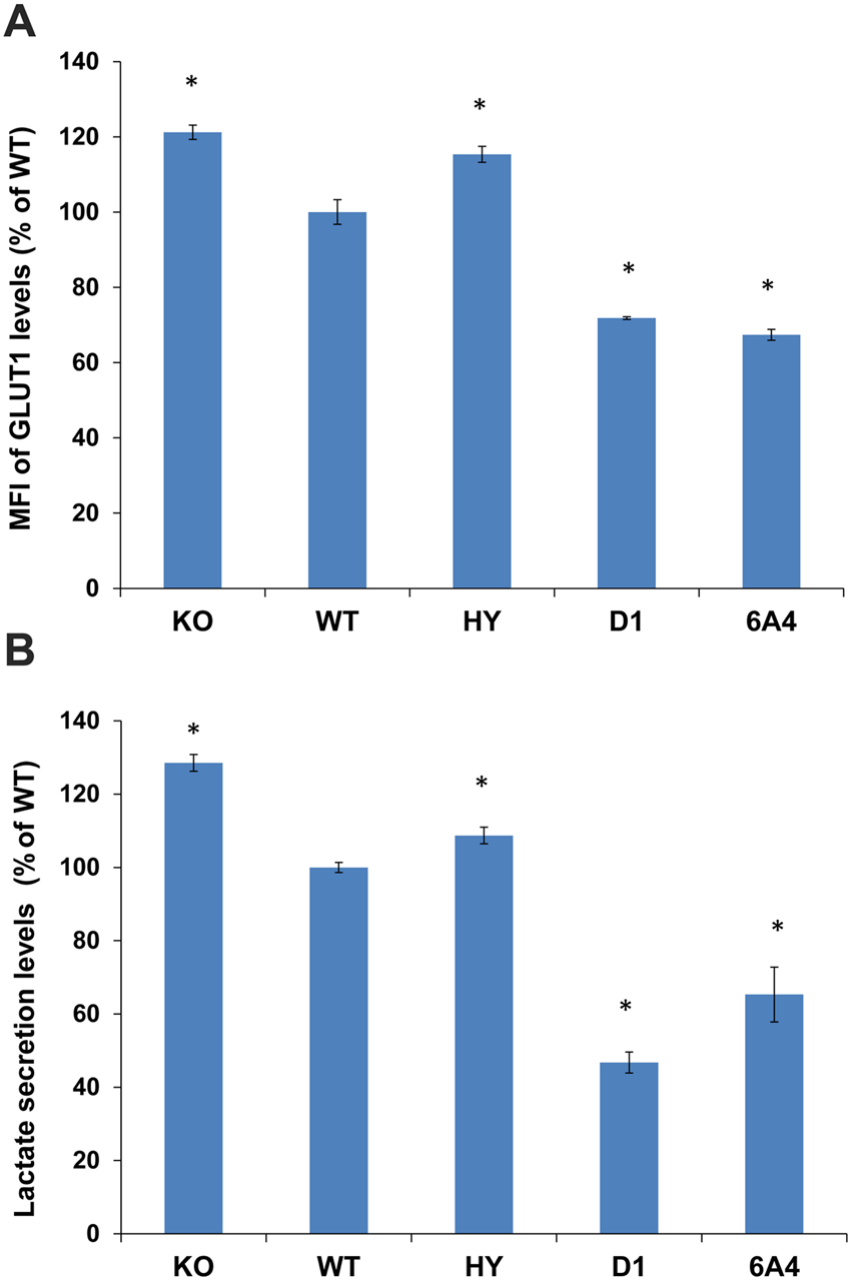
Glucose Metabolism of D1 and 6A4 SIRT6 mutants is enhanced relative to the WT. (**A**) Analysis of the expression level of GLUT1 on cells. KO MEFs expressing the different SIRT6 variants were analysed by flow cytometry to measure the level of GLUT1 on the cell membrane. The mean fluorescent units (MFI) of the flow cytometry signal of all mutants were normalized to the WT level. (**B**) Lactate secretion levels from the MEF cell lines expressing the different SIRT6 variants were obtained by measuring NADH formation following incubation with lactate dehydrogenase. All results are normalized to the WT lactate secretion level. The data presented is the average of three independent technical repeats of each experiment while the error bars represent the standard deviation from the average. Statistically significance differences of the different SIRT6 variants or the KO from the WT MFI (**A**) or lactate levels (**B**) are labeled with black star (p < 0.05). The expression level of the different SIRT6 variants is very similar and is shown in Fig. 4A.

**Figure 6:**
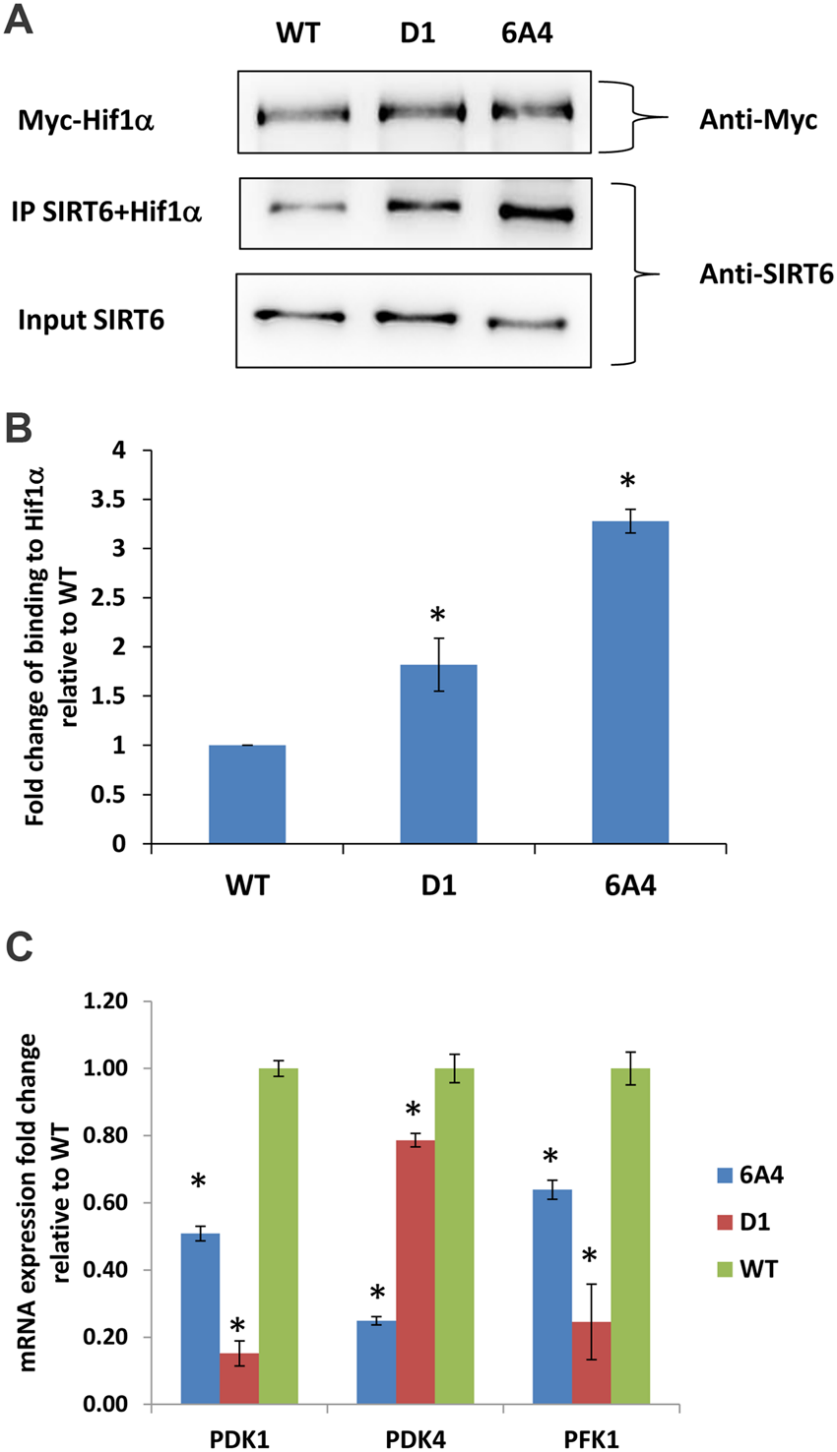
Analysis of D1 and 6A4 interaction with Hif1α and inhibition of gene expression relative to WT SIRT6. (**A**) IP analysis of SIRT6-Hif1α interaction. Extracts from HEK293 cells expressing Hif1α were IP with α-Myc antibodies conjugated to agarose beads followed by incubation with purified SIRT6 mutants or WT. Western blot analysis of the input of SIRT6, the IP SIRT6 and the levels of Hif1α are shown indicating higher interaction of D1 and 6A4 with Hif1α. (Original blots are shown in Supplementary Fig. S11) (**B**) Quantification of binding of SIRT6 to Hif1α is based on band intensity of western blot analysis using Image J software (Fig. 6A and an additional independent experiment). The levels of bound SIRT6 to Hif1α were quantified in the IP sample of D1 and 6A4 relative to the input levels (based on the bands intensity) the results were then normalized to the levels obtained for the WT SIRT6 samples. Error bars represent standard deviation of two independent biological repeats (p < 0.05). (**C**) Inhibition of PDK1, PDK4 and PFK1 gene expression by D1 and 6A4 relative to WT SIRT6. Gene expression analysis was performed by real-time PCR with primers that are specific to each gene. The data is the average of three independent biological repeats while the error bars represent the standard deviation from the average. Statistically significance differences relative to WT SIRT6 are labeled with black star (p < 0.01).

Next, we examined the phenotype of cells expressing D1 and 6A4 exhibiting normal or low deacetylation activity but high deacylation activity relative to the WT protein. Previous studies have shown that SIRT6 regulates glucose homeostasis by deacetylation of H3K9ac at the promoters of Hif1α target genes thereby repressing glucose uptake and absorbance in cells^31^. This phenotype is mediated by several changes in cell physiology including reduced levels of the glucose transporter, GLUT1, on the outer cell membrane, reduced expression of glycolysis related genes and decreased lactate secretion^31^. To test the effect of our mutants on glucose metabolism, we measured the expression of GLUT1 on the cell membrane and lactate levels in the medium of MEFs that stably express the different SIRT6 variants (Fig. 4A). As expected, the expression of GLUT1 on the outer membrane of cells expressing WT SIRT6 was lower realtive to cells expressing the inactive H133Y mutant or cells containing SIRT6 KO. In contrast, the expression of GLUT1 in D1 and 6A4 cells was significantly lower relative to cells expressing the WT, demonstrating improved glucose metabolism phenotype of cells expressing D1 and 6A4 (Fig. 5A). Consistently, we measured a significant decrease in the lactate levels secreted by cells expressing D1 and 6A4 relative to cells expressing the WT protein (Fig. 5B). Previously, SIRT6 was shown to interact with Hif1α to modulate its gene suppression activity in cells^31^. To examine whether the improved glucose metabolism phenotype in cells expressing the D1 and 6A4 mutant is associated with increased interaction of the SIRT6 mutants with Hif1α, we performed immunoprecipitation (IP) experiments. To test the level of SIRT6-Hif1α interaction, we expressed a Myc-tagged Hif1α in HEK293 cells and captured Hif1α using agarose beads coated with α-Myc antibodies. Next, equal amount of purified WT SIRT6, D1 and 6A4 variants were incubated with the beads and the amount of captured SIRT6 was analysed by western blot analysis. We clearly observed that the two SIRT6 mutants exhibit higher binding to Hif1α in correlation with their effect on glucose metabolism phenotype in cells (Fig. 6AB and Supplementary Fig. S12). To examine whether SIRT6 mutants exhibit higher suppression of glycolytic gene expression, we used real-time PCR analysis of selected glycolytic genes in MEFs expressing WT SIRT6, D1 and 6A4 variants. In agreement with higher interaction with Hif1α, we observed a significantly stronger inhibition of PDK1, PDK4 and PFK1 gene expression (Fig. 6C). Finally, we examined the effect of these mutants on the repression of Hif1α target genes using the previously reported luciferase assay^31^. This assay is based on a plasmid containing luciferase reporter gene controlled by three repeats of Hypoxia-Response Elements (HRE). Thus, we co-transfected SIRT6-KO cells with HRE-Firefly luciferase plasmid, T7 Renilla (for transfection efficiency) and an empty vector, SIRT6 WT, 6A4, D1 or Hif1α. Hif1α overexpression activates the expression of luciferase, confirming that these HRE elements responds to Hif1α (Supplementary Fig. S13). Using this assay, we found that cells which were co-transfected with SIRT6 WT, 6A4 or D1 mutants had reduced luciferase expression, relative to an empty plasmid. These results show that these SIRT6 variants can repress Hif1α target genes even in the absence of deacetylation activity in the case of 6A4 mutants (Supplementary Fig. S13).

## Discussion

The directed evolution of SIRT6 for improved deacylation activity enabled the generation of two mutants, D1 and 6A4, with significantly enhanced activity. Kinetic analysis of these mutants indicates that improvements in catalysis stems from enhanced k_cat_ suggesting that the mutations in D1 and 6A4 improves the acyl-transfer efficiency rather than the binding of the peptide or the NAD^+^ moieties. In accordance, the mutations identified in these variants are located at the vicinity of the acyl moiety and NAD^+^ binding site and thus, can possibly lead to better positioning of the substrate and NAD^+^ for acyl-transfer catalysis (Fig. 2). The improvements in catalytic activity obtained in the selected mutants are relatively low in comparison to other enzymes evolved by directed evolution^14,24^. These relatively small improvements can stem from the high sequence conservation of the sirtuins from different organisms. In accordance, structural analysis of the sirtuin deacetylase domain from different organisms reveals high structural conservation including the maintenance of the Rossman fold and zinc-binding domain over very large evolutionary time scales^32^.

Interestingly, mutations in D1 and 6A4 led to significant improvements in deacylation activity with either no effect or a dramatic reduction in deacetylation activity, respectively. Recent study has shown that G60A mutation leads to a dramatic reduction of SIRT6 deacetylation activity with minor decrease in deacylation activity allowing investigating the specific effects of SIRT6 deacylation in cells. This study revealed many new potential SIRT6 substrates that are secreted from cells due to SIRT6 deacylation activity. This study, however, did not report the effect of SIRT6 G60A mutation on glucose homeostasis. Sequence analysis of the D1 and 6A4 mutants discovered here revealed the presence of the H68S mutation located at the NAD^+^ binding loop that is located in relatively close proximity to the G60 position. Kinetic analysis of D1 and 6A4 at increased NAD^+^ concentrations and saturated levels of TNF-α K20myr peptide revealed a decreased affinity for NAD^+^ (increased K_M_, Supplementary Figs S6–7) suggesting a negative effect of the H68S mutation in accordance with its location at the NAD^+^ binding loop. However, given that the physiological concentration of NAD^+^ is estimated to be ~300 μM^33,34^ the decrease in NAD^+^ affinity should not affect D1 and 6A4 activity in cells.

Previously, SIRT6 was shown to exhibit mono ADP-ribosylation activity on PARP1 leading to enhanced genome stability^12,35^. We cannot exclude the possibility that D1 and 6A4 exhibit improved cellular activity due to improved ADP ribosylation, however, this is unlikely given the higher K_M_ of these mutants to NAD^+^. Examination of the activity of D1 and 6A4 variants in cells relative to the WT activity shows an improved glucose metabolism indicated by a decrease in GLUT1 expression and lactate production (Fig. 5). The improved glucose metabolism in cells expressing D1 and 6A4 was associated with increased interaction with Hif1α and the inhibition of expression of glycolytic genes (Fig. 6). Previously, glucose metabolism was directly related to SIRT6 deacetylation of H3K9Ac on promoters of glycolytic genes. Our results showing that the cellular expression of 6A4 mutant, exhibiting a very low deacetylation activity on H3K9Ac (Fig. 4), leads to improved glucose metabolism suggest that additional molecular pathways can affect glucose metabolism levels in cells. Our data suggests that these pathways can be activated/inhibited due to SIRT6 deacylation activity. Given the large amount of potential new SIRT6 acylated substrates recently identified^13^, it is possible that SIRT6 deacylation of one of these substrates can lead to increased interaction with Hif1α and enhanced glucose metabolism phenotype. Since 6A4 mutant exhibits increased K_M_ for acylated substrates (Table 1), the concentration of potential acylated substrates of SIRT6 could be high (e.g. above 20 μM) to allow maximal activity of 6A4 in the cell. Given the efforts to develop small molecules that will lead to SIRT6 activation, our results suggest that screening for small molecules that lead to enhanced SIRT6 deacylation activity may lead to beneficial cellular phenotype. In addition, the rapid advancement of CRISPR/CAS9 technology may enable the insertion of the beneficial mutations identified in D1 and 6A4 into mice or other model organisms to enhance the endogenous SIRT6 activity for future animal model studies.

In summary, in this work we isolated two SIRT6 mutants with increased deacylation activity but with no effect or even reduced deacetylation activity on H3K9Ac and H3K56Ac in cells. The separation of function in SIRT6 between deacylation and deacetylation highlight the mechanistic difference between these two activities and allowed us to examine the specific effect of increasing deacylation activity on cellular metabolism. Interestingly, we found that such specific increase in SIRT6 deacylation activity leads to improved glucose homeostasis maintenance in cells. Given the search for small molecules that will serve as SIRT6 activators^36^, our data suggests that screening for small molecules that lead to improved SIRT6 deacylation activity can be beneficial for  SIRT6 mediated cellular processes. In addition, our data suggests that glucose homeostasis can be improved by SIRT6 deacylation of still unknown cellular proteins regardless of its deacetylation activity.

## Materials and Methods

### Generation of SIRT6 ancestral library plasmids

pcDNA3.1-hSIRT6 was obtained from Prof. Haim Cohen’s lab and used as a template to amplify hSIRT6 gene by standard PCR reaction. Next, 20 µg of hSIRT6 PCR products were incubated in 250 µl DNAseI reaction mixture: 4 U DNaseI (NEB), 10 × DNaseI buffer and 10 mM MnCl_2_ for 2 min at 15 °C. Reaction was quenched with EDTA at final concentration of 25 mM, on ice. Products were run on 2.5% agarose gel and ~150 bp fragments were cut from the gel, extracted and purified using GeneJET gel extraction kit (Thermo). Oligonucleotides containing the desired mutation (Table S1) were incorporated into hSIRT6 sequence by a primerless PCR reaction using Bio-x-act DNA polymerase (Bioline) reaction. Reaction products were then diluted 1:10,000 and served as a template for nested PCR using KOD polymerase (Merck) to amplify the full length *hSIRT6* gene. The resulting PCR fragments were incubated with DpnI (Thermo) for 30 min at 37 °C, purified and cloned into pMAL C2 vector using T4 DNA ligase (NEB). Products were transformed into DH5α and plated on LB agar containing 100 µg/ml Ampicillin. Colonies were pooled together and library plasmids were purified using Wizard® Plus SV Miniprep (Promega).

### Screening of SIRT6 Ancestral library

The ancestral library was transformed into *E. coli* Rosetta 2 strain. Single colonies were picked and grown in 96 deep-well plates containing 600 µl of TB and induced at OD_600_ of 0.6 with 0.1 mM IPTG for 16 hrs at 18 °C. Individual cell pellets were resuspended in 100 µl lysis buffer ((BugBuster®, MILLIPORE), 1:1000 protease inhibitor cocktail (Calbiochem), 50 mM Tris pH 7.4 and 100 mM NaCl) and incubated with shaking at 25 °C for 1 hour, followed by 20 min centrifugation at 4000 g at 4 °C. The activity of lysates was measured by Fluor De Lys (FDL) assay^27^ using 100 µM TNFα K20myr peptide fused with 7-amino-4-methylcoumarin fluorescent group (AMC) to the c-terminus (see below) and 0.5 mM NAD^+^.

### Expression and Purification of Recombinant SIRT6 in E.coli

pMAL plasmids containing the gene of SIRT6 variants were transformed into Rossetta *E. coli* competent cells. Cells were grown in TB medium containing ampicillin and chloramphenicol at 37 °C to OD_600_ of 0.6 and induced with 0.1 mM IPTG followed by 16 hours incubation at 16 °C. Cells were lysed by French Press in lysis buffer (20 mM Tris-HCl pH 7.5, 100 mM NaCl, and EDTA free-protease inhibitor cocktail (Calbiochem). Cell debris were removed by centrifugation at 16,000 × g at 4 °C for 40 min. The supernatants were loaded onto a pre-equilibrated amylose column eluted with elution buffer (20 mM Tris-HCl, 100 mM NaCl and 20 mM maltose at pH 7.5). The purity of the proteins was assessed by SDS-PAGE. The protein concentration was defined according to the Bradford method using bovine serum albumin as the standard.

### Fluor de lys (FDL) activity assay

TNFα peptide K20myr conjugated to AMC group at the carboxyl terminus (Peptron) was used for the FDL screening assay. FDL activity assay was performed as describe before^27^ with minor modifications. Briefly, purified SIRT6 WT or improved mutants were incubated at 37 °C in reaction buffer containing 5 mM NAD + and 0.1 mM TNFα K20myr-AMC peptide in Assay buffer (50 mM Tris-HCl pH 8.0, 137 mM NaCl, 2.7 mM KCl, 1 mM MgCl_2_ and 1 mg/ml BSA). 50 µl aliquots were taken in 5 min intervals and mixed with 50 µl of the developer solution (assay buffer + 50 µM HCl pH 8, 3 mg/ml trypsin and 10 mM NAM). The quenched samples were kept at 37 °C for 20 min prior to fluorescence reading. Fluorescence readings were obtained using the TECAN infinite series 200 fluorimeter with the excitation wavelength set to 360 nm and the emission set to 460 nm in black 96-Well plates (Greiner).

### Cell culture and antibodies

The human embryonic Kidney (HEK)293T cell line was obtained from American Type Culture Collection. *SIRT6* knock out (KO) MEFs were obtained as a kind gift from Prof. Haim Cohen, Bar Ilan University. All cells were grown in Dulbecco’s modified Eagle’s medium supplemented with 10% FBS, 100 U/ml penicillin, 100 µg/ml streptomycin and 2 mM L-Glutamine (all purchased from Biological Industries, Israel) and maintained in a 37 °C incubator with 5% CO_2_. Primary antibodies against SIRT6, Histone 3, β-actin, H3K9ac, H3K56Ac, GLUT1 and secondary antibodies (goat anti mouse HRP, goat anti rabbit HRP and goat anti rabbit Alexa488) were all purchased from Abcam. Antibody against Flag used for SIRT6-FLAG was purchased from Sigma.

### Generation of SIRT6 KO cell line

*SIRT6* KO 293T cells were generated using CRISPR-Cas9 gene disruption as previously described^37^ with minor modifications. Lentiviruses containing plasmid encoding guide RNA toward SIRT6 were produced in 293T cells and used to transduce 293T cell line. Virally transduced cells were diluted to 1 cell/200 µl and transferred to 96 wells plate containing 1 µg/mL puromycin (Sigma). Colonies were then trypsinised and re-plated in 12 wells plate and the absence of SIRT6 was verified by western blot.

### Transient and stable expression of SIRT6 variants

For transient transfection, WT hSIRT6, D1 and 6A4 variants were cloned into pcDNA3.1 + vector fused to a FLAG tag. Trans IT-LT1 (Mirus) was used to transfect HEK 293T cells according to the manufacturer’s protocol. For stable expression, SIRT6-FLAG variants were cloned into pWZL-hygro retroviral vector and co-transfected into 293T using retroviral gag pol and VSV helper vectors. Retroviruses were collected from the medium and used to transduce MEFs SIRT6 KO cell line. Virally transduced cells were selected by the addition of 200 µg/mL of Hygromycin B (Gold Biotechnology).

### Lactate Assay and GLUT1 staining

*SIRT6* KO MEFs transduced with WT SIRT6-FLAG or SIRT6 mutants were grown in DMEM to 50% confluency. Medium was collected and treated with 7% perchloric acid (PCA) followed by centrifugation at 12000 RPM for 10 min at 4 °C. Samples were incubated for 30 min at 37 °C in reaction buffer containing 300 mM glycine, 250 mM hydrazine sulfate, 5 mM EDTA, 630 mM NaOH, 4.5 mM NAD + and 5 U lactate dehydrogenase (SIGMA). Absorbance of each sample was measured at 340 nm. For determination of GLUT1 levels on the outer cell membrane, the same cells were harvest, washed with PBS and fixed with 2% paraformaldehyde. Then, cells were incubated with rabbit anti-GLUT1 antibody for 1 hr at 25 °C, washed and incubated with goat anti-rabbit Alexa 488 secondary antibody for 1 hour in the dark. Cell were washed again, resuspended in PBS buffer containing 3% BSA, 1% sodium azid and taken to FACS analysis.

### HPLC kinetic Assay

SIRT6 variants at a concentration of 0.3 µM were incubated in a reaction buffer containing 50 mM Tris-HCl pH 7.4, 137 mM NaCl, 1 mM MgCl_2,_ 3 mM KCl, 0.5 mM NAD + for 30 min at 37 °C. Reaction was stopped using cold stop solution: 0.2 M HCl, 0.32 M Acetic acid in 50% methanol and neutralized by 0.3 M K_2_CO_3_. Samples were centrifuge 17,000 g for 10 min at 4 °C. Supernatants were transferred to HPLC vials and run on HPLC using Kinetex C18 column (100 A, 100 mm × 4.6 mm, 2.6 µm, Phenomenex) at 214 nm. Product and substrate area peaks were quantified to derive the initial rate at each peptide concentration and kinetic parameters were determined by fitting the initial velocity values at different substrate concentration to the Michaelis-Menten equation.

### Chromatin fractionation and deacetylation assay

The chromatin was purified from HEK 293T SIRT6 KO cell line. The cell were collected, washed with PBS, re-suspended with lysis buffer (10 mM HEPES pH 7.4, 10 mM KCl, 0.1% Triton X-100 with protease inhibitor) and incubated for 20 minutes on ice. The lysate was centrifuged at 4 °C for 10 minutes to separate the supernatant containing the cytoplasmatic proteins and the pellet containing the nuclei. The nuclei was washed once with the lysis buffer, re-suspended with low salt buffer (10 mM Tris-HCl pH 7.4, 0.2 mM MgCl_2_, 1% Triton-X and protease inhibitor) and incubated for 15 minutes on ice. Following centrifugation for 10 minutes at 4 °C, the supernatant containing the nucleoplasmatic proteins and the pellet containing the chromatin were separated. The chromatin fraction was re-suspended in HCl 0.2 N and incubated for 20 minutes on ice. After centrifugation of 10 minutes at 4 °C the supernatant was kept in the same volume of 1 M Tris-HCl pH 8 buffer. The deacetylation assay was performed on the chromatin fraction with purified SIRT6 mutants at room temperature. To stop the reaction at different time points aliquots were removed at times 0, 60, 120 and 210 min into sample buffer and incubated for 5 minutes at 99 °C. The overall deacetylation assay contains 70 µl of histones, 40 µl of purified SIRT6, 20 µl of 125 mM NAD + and 20 µl of deacetylation buffer (30 mM Tris-HCl pH 8, 4 Mm MgCl_2_ and 1 mM DTT).

### Immunoprecipitation (IP) of SIRT6 with Hif1α

HEK 293T cells were transfected with pcDNA Myc-Hif1α. Next, 16 hours post transfection proteins were extracted using lysis buffer (150 mM KCl, Tris HCl pH 7.5 50 mM, NP-40 1%, DTT 0.5 mM and protease inhibitors). In parallel, Myc-tag beads (Merck) were blocked with blocking solution (PBS + 5% goat serum). The blocked beads were incubated 2 hours with the different protein extracts and washed twice with lysis buffer. The beads were then washed twice with wash buffer (KCl 150 mM, Tris HCl pH 7.5 25 mM, 5% glycerol, 1 mM DTT, 0.1% Triton X-100, 0.2 mM EDTA, and protease inhibitors. The beads were then divided to three tubes and incubated over night at 4 °C with purified WT SIRT6, D1 and 6A4 at a concentration of (2 μg/ml) in a volume of 500 μl. The proteins were then eluted from the beads with lameli 2 × sample buffer (100 μl) and incubated 10 min at 95 °C.

### Real-Time PCR analysis

KO MEFs overexpressing the different SIRT6 variants were grown in rich DMEM and harvest at 40–60% confluency. Total RNA was isolated using RNA PureLink mini kit (Invitrogen). Each RNA sample was used as a template for synthesis of single strand cDNA for detection of different target gene of the TF by reverse transcriptase (Thermo) using oligo-dT as primer. Relative transcript levels were then determined by real-time PCR analysis using a 7300 Applied Biosystem machine. The reaction mix contained 10 µl of SYBR green mix (Applied Biosystem), 10 µM of primers, 2 ng/µl cDNA and DDW to total reaction volume of 20 µl. Actin was used as the internal standard.

### Luciferase reporter assay

HRE-luciferase plasmid was kindly provided by Mostoslavsky laboratory and the procedure was performed as previously reported^31^ with minor modifications. In brief, HEK 293T SIRT6 KO cells were plated in 50% confluence. These cells were then transfected with Renilla-null (10% of total plasmid mass) and HRE-Firefly (45% of total mass) plasmids, as well as one of the following plasmids: SIRT6 WT, 6A4, D1, HIF1α or an empty backbone. Transfections were performed according to manufacturer protocol for PolyJet reagent. Dual Luciferase kit was used to measure Firefly and Renilla luciferases. Firefly results were normalized by Renilla expression. Each experiment was done in biological duplicates in addition to technical triplicates for the luciferase assay.

## Electronic supplementary material

Supplementary Information
