# Supplementary material for: Directed evolution of SIRT6 for improved deacylation and glucose homeostasis maintenance
Source: Sci Rep. 2018 Feb 23;8:3538. doi: 10.1038/s41598-018-21887-9 (PMC5824787; doi:10.1038/s41598-018-21887-9)
Supplement: Supplementary file 1 — Supplementary Information [file 41598_2018_21887_MOESM1_ESM.docx]

**Supplementary Information**

**Directed evolution of SIRT6 for improved deacylation and glucose homeostasis maintenance**

Or Gertman^1^, Dotan Omer^1,2^, Adi Hendler^1^, Daniel Stein^1^, Lior Onn^1^, Yana Khukhin^1^, Miguel Portillo^1^, Raz Zarivach^1^, Haim Y. Cohen^3^, Debra Toiber^1^ and Amir Aharoni^1,4*^

^1^Department of Life Sciences, Ben-Gurion University of the Negev, Be’er Sheva 84105, Israel. ^2^Current address: Smartzyme Innovation LTD, Ilan Ramon, Science Park-Ness Ziona, Israel. ^3^The Mina & Everard Goodman Faculty of Life Sciences, Bar-Ilan University, Ramat-Gan 5290002, Israel. ^4^The National Institute for Biotechnology in the Negev, Ben-Gurion University of the Negev, Be’er Sheva 84105, Israel.

^*^ Correspondence should be addressed to A.A. (aaharoni@bgu.ac.il)


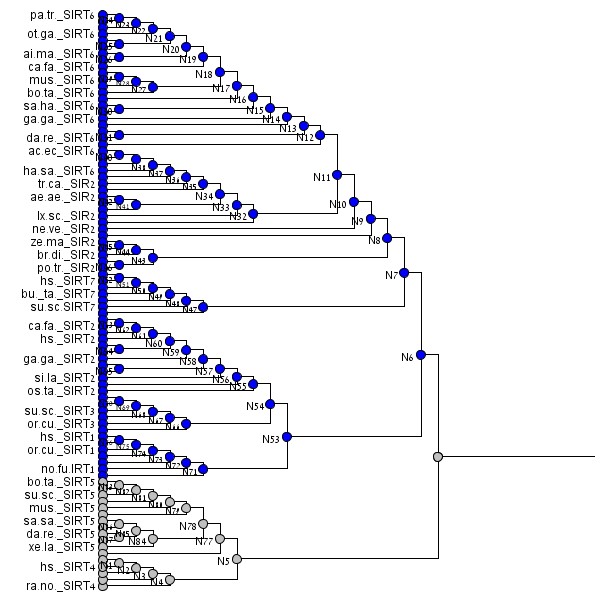


**Fig. S1**: Phylogenetic tree of the sirtuins that was used for the prediction of the ancestral sequences at the different nodes. The most probable ancestor at Node 6 (N6) was chosen for the library design.


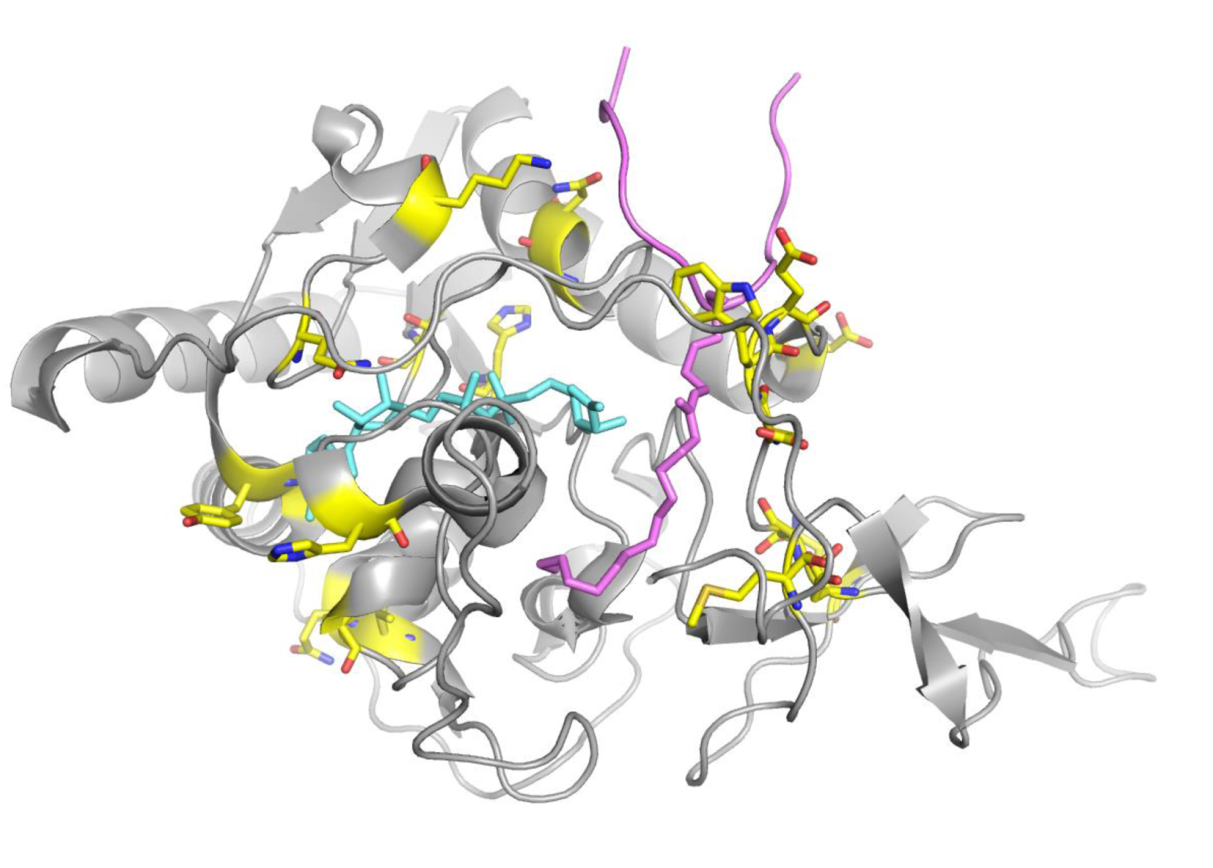


**Fig. S2**: Location of the positions mutated in the SIRT6 library highlighted on SIRT6 crystal structure (3ZG6). All positions are highlighted in yellow in a stick representation. The bound peptide and the ADPR (the NAD analogue) are highlighted in violet and magenta, respectively. All residues are located 15Å from the catalytic histidine and are distributed around the active site. The exact positions are listed in **Table S1**.


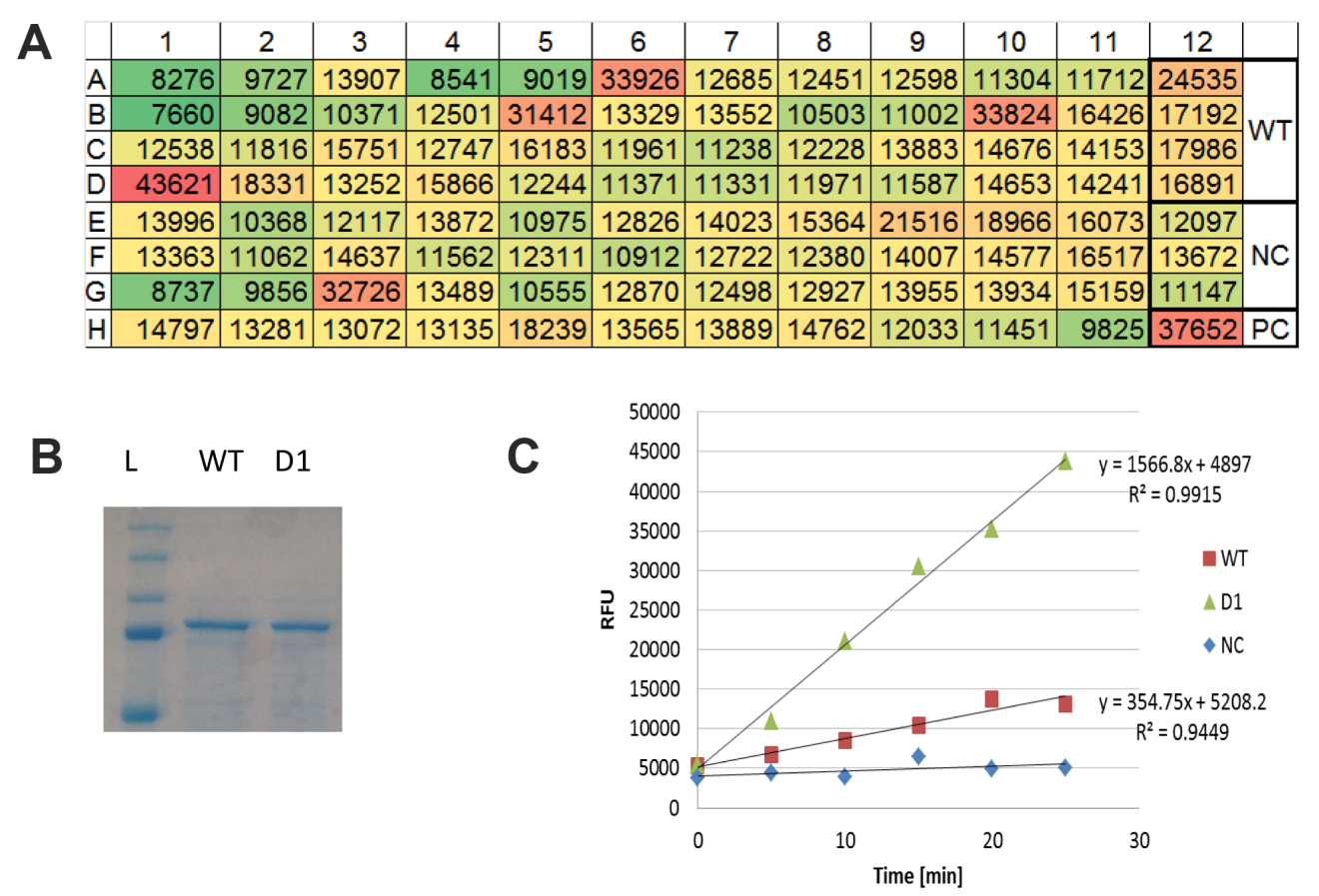


**Fig. S3**: A representative steps in the screening process for the identification of improved SIRT6 mutants. (**A**) A representative screening of 88 SIRT6 mutants using the Flour de Lys assay. SIRT6 mutants were tested for deacylation activity using the TNF-α peptide fused to AMC to facilitate the FDL detection. Reactions were stopped following 30 min of incubation with the TNF-α substrates. Improved mutants were identified by higher fluorescence signal relative to the WT. (**B-C**) SDS PAGE gel of the WT and D1 variant purified on a small scale for the verification of improvements in specific activity. The activity of the purified WT and D1 was then tested by FDL at different time points. D1 exhibits a significant improvement in reaction rate with the FDL substrate relative to the WT.


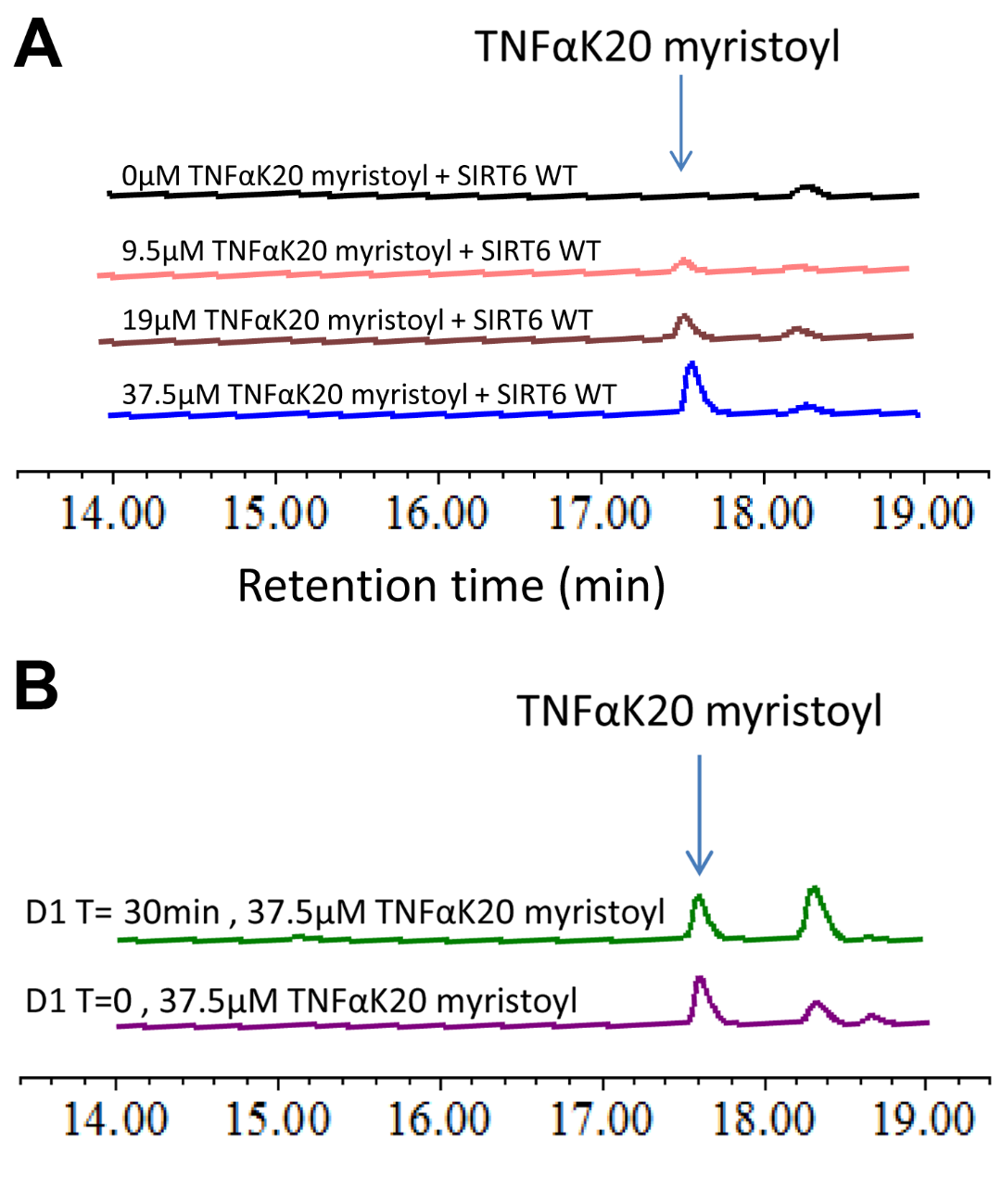


**Fig. S4**: Representative high-performance liquid chromatography (HPLC) traces of TNFαK20myristoyl. (**A**) Traces showing TNFαK20myristoyl at different concentrations at time 0 following the addition of WT SIRT6, a clear increase in TNFαK20myristoyl peak intensity (highlighted with an arrow) is observed at increased peptide concentration. (**B**) A small decrease in the peak intensity is observed at high peptide concentration following incubation of 30 min with WT SIRT6.


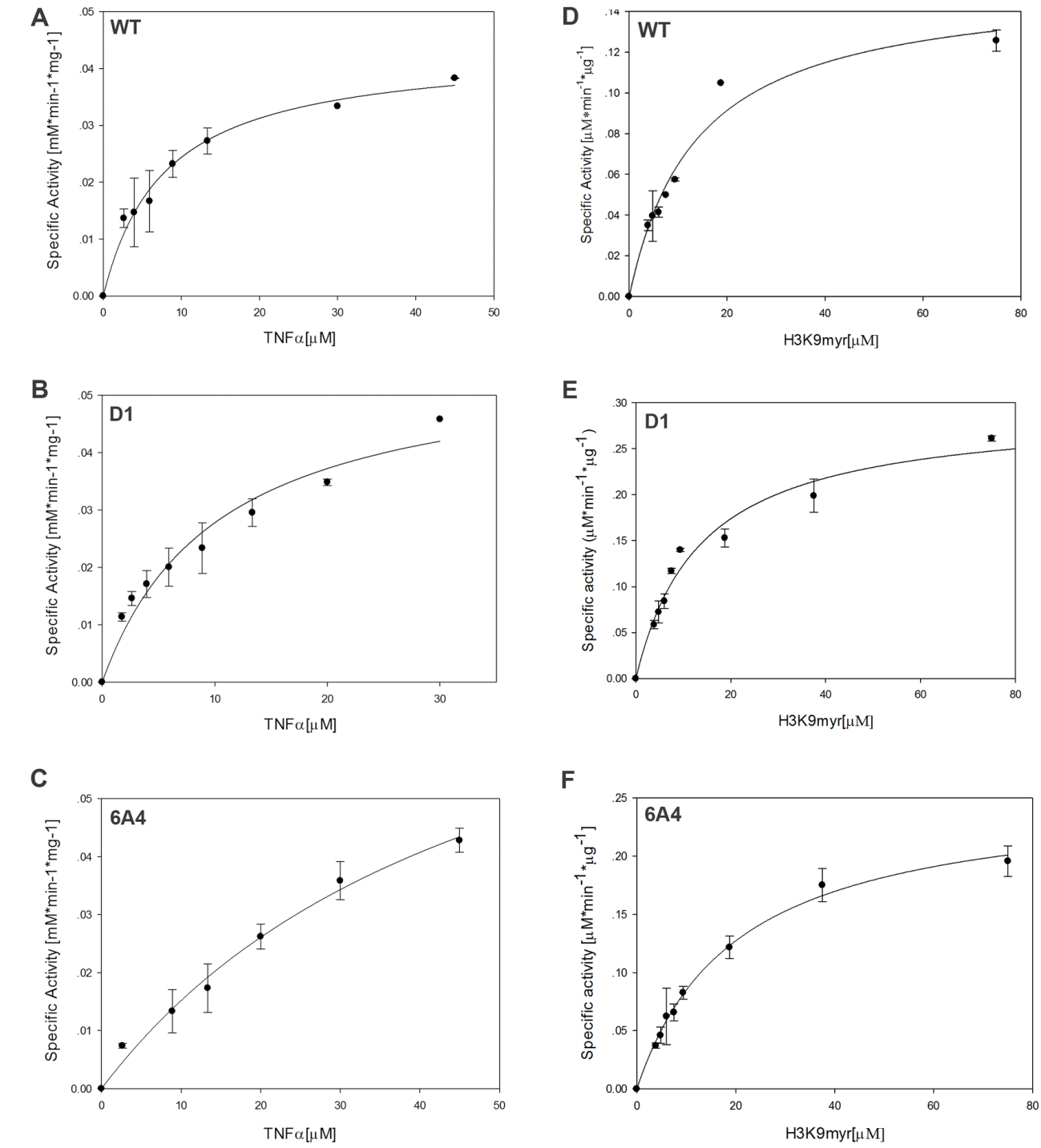


**Fig. S5:** Michaelis Menten (MM) analysis of the different SIRT6 variants including WT, D1 and 6A4. Initial rates were measured following incubation of the SIRT6 variant with different TNF-α K20myr peptide (**A-C**) or H3K9myr peptide (**D-F**) at different concentrations. The initial rates were translated to specific activity and plotted against substrate concentration and fitted to the MM equation to derive the k_cat_ and K_M_ parameters reported in **Table 1** (main text). Each experiment was performed in three biological repeats and representative experiment is shown.





**Fig. S6**: Michaelis Menten (MM) analysis of the different SIRT6 variants including WT, D1 and 6A4. Initial rates were measured following incubation of the SIRT6 variant with different NAD+ concentrations at saturated TNF-α K20myr peptide concentration of 50μM.


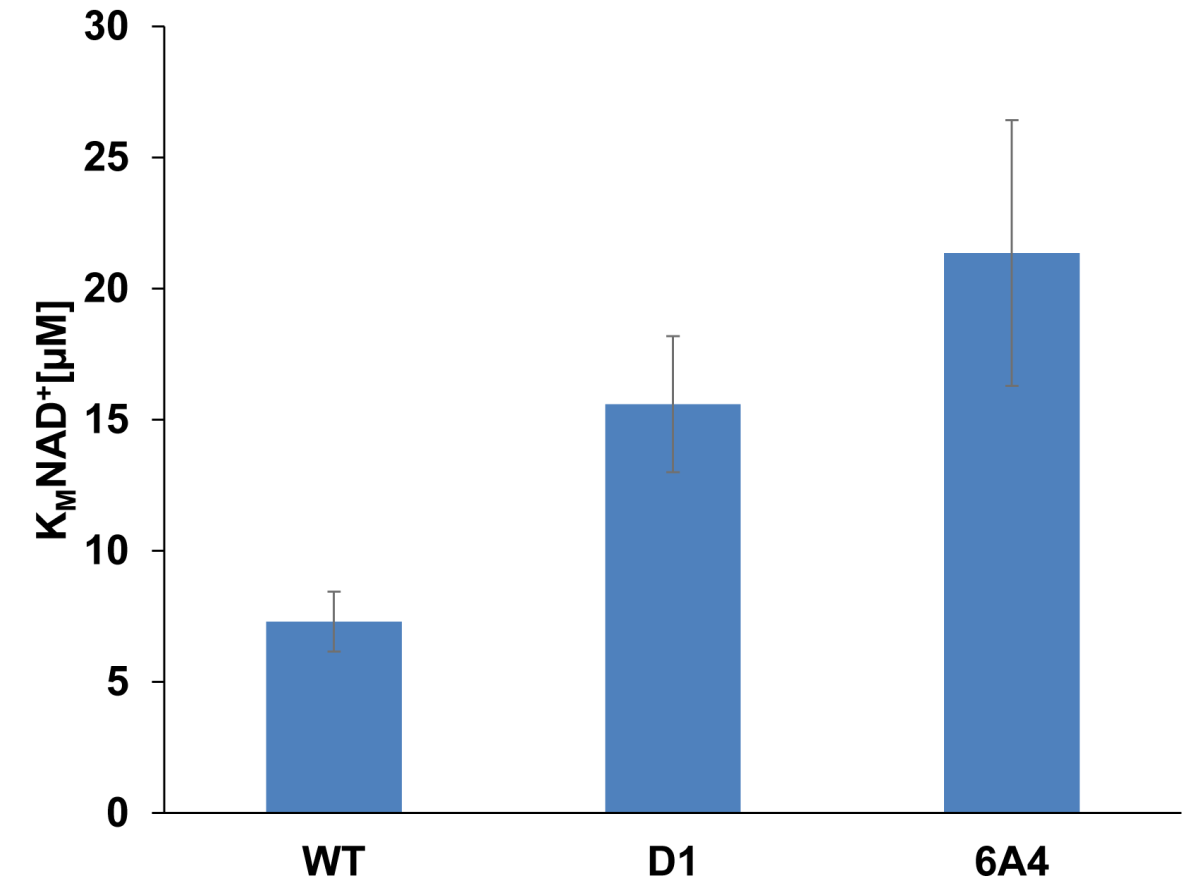


**Fig. S7:** K_M_ values of WT SIRT6, D1 and 6A4 variants for NAD^+^. The values were derived from Michaelis-Menten analysis of the rate of TNF-α K20myr deacylation measured at saturated peptide concentration of 50μM. The data is the average of three independent biological repeats while the error bars represent the standard deviation from the average. Statistically significance differences relative to WT SIRT6 are labeled with black star (p<0.01).





**Fig. S8**: Generation of HEK293 cell line that contains *SIRT6* knock-out (KO). (**A**) SIRT6 KO cell line was generated using standard CRISPR/CAS9 technology (see detailed description in Materials and Methods section). *SIRT6* KO cell line (CR35) was chosen by the lack of SIRT6 expression and the high level of H3K56ac. (**B**) Original western blot CR34 was not utilized in this study due to inconsistency between Sirt6 expression and H3K56Ac level.





**Fig. S9**: Original western blots for the analysis of SIRT6 deacetylation of H3K9Ac and H3K56ac in MEFs shown in **Fig. 4** main text. Mutant Mx (shown in **A**) is a non-relevant SIRT6 mutant that was omitted from that analysis shown in the main text. Analysis was performed on crude cell lysates prepared from equal amount of KO MEFs cells that stably express the different SIRT6 variants, including WT, D1, 6A4 and the non-catalytic H133Y (HY). (**B**) Original H3 western blot analysis no other bands were visible on the full gel blot analysis.


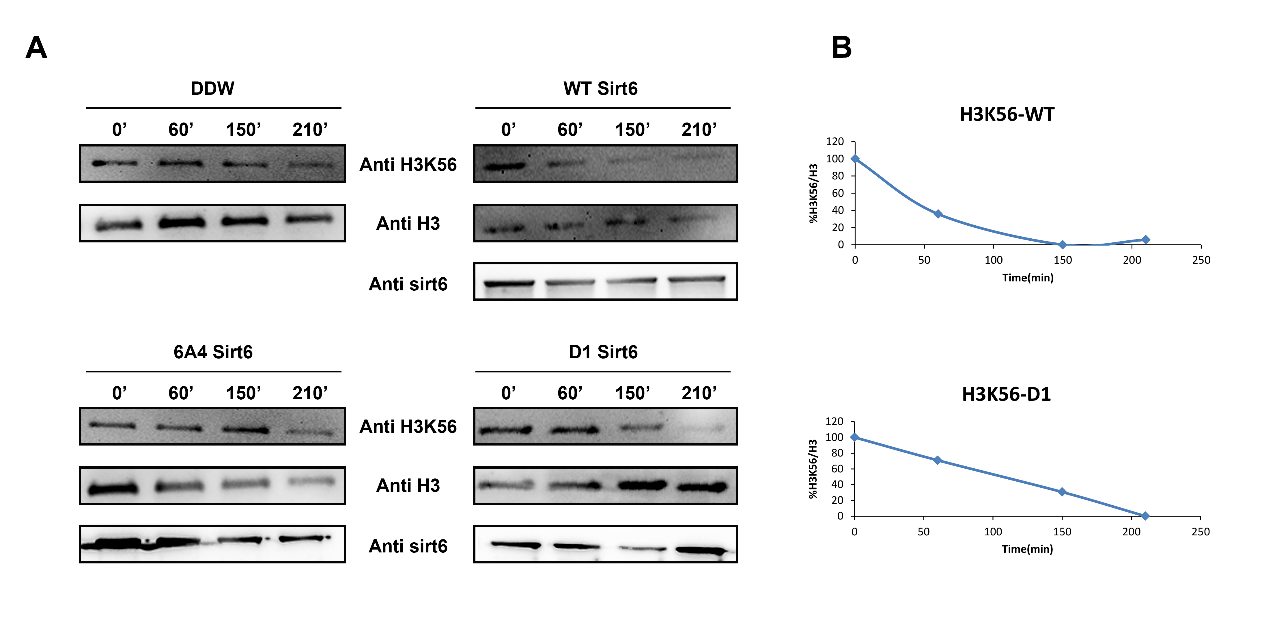


**Fig. S10**: Western blots for the kinetic analysis of SIRT6 deacetylation of H3K56Ac in the chromatin fraction isolated from HEK293 cells of CR35 cell line (**Fig. S6**). (**A**) Blots showing the analysis at 4 different time points of WT SIRT6, D1, 6A4 and DDW as a control. (**B**) Quantification of deacetylation activity normalized to time 0, presenting the percentage of H3K56Ac/H3 signal quantified by image J. Only WT and D1 mutants exhibit detectable deacetylation activity.


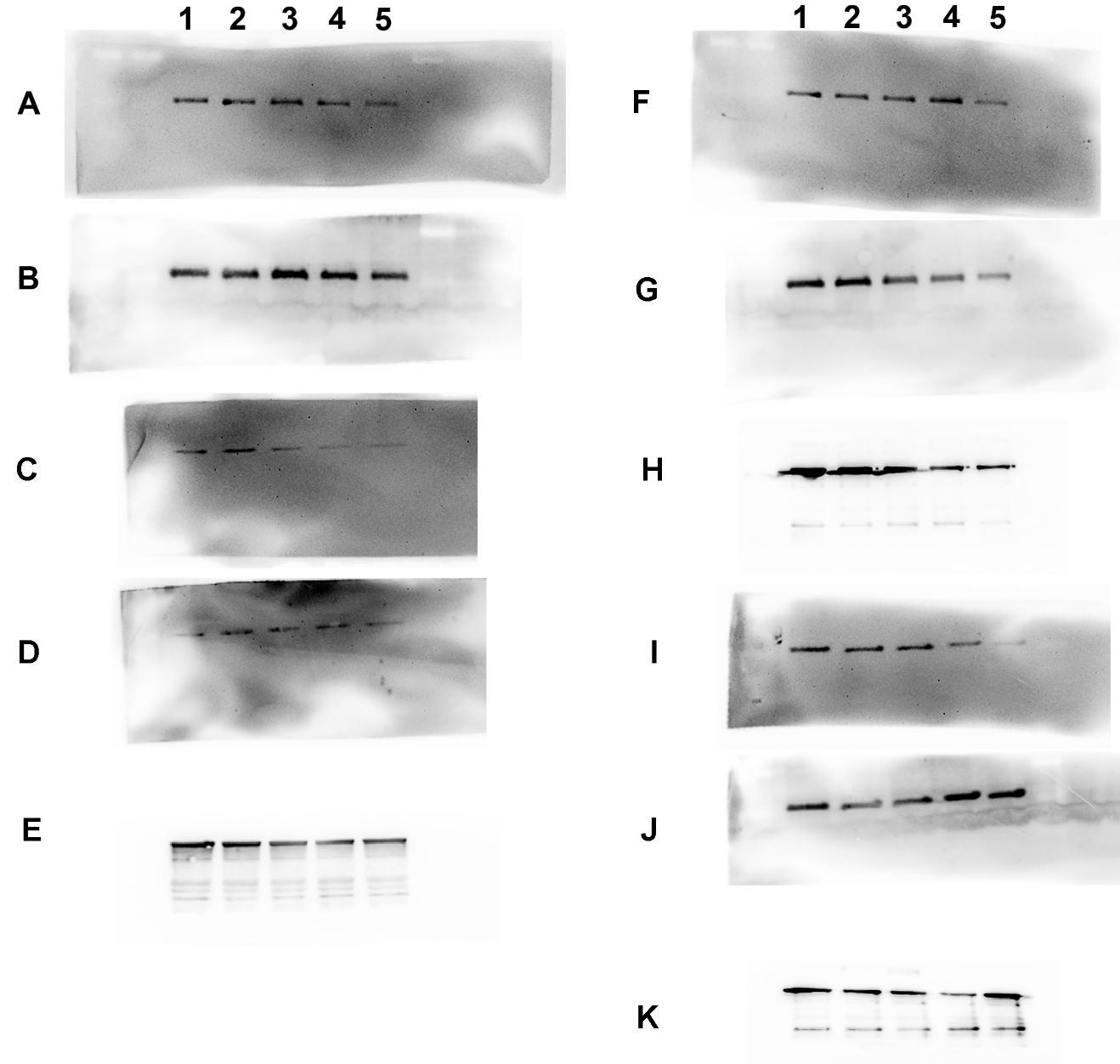


**Fig. S11**: Original western blots for the kinetic analysis of SIRT6 deacetylation of H3K56Ac shown in Fig. S10. Sample 2-5 correspond to time 0, 60, 150 and 210 min respectively. Sample 1 was taken before reaction equilibration and thus was taken out from the analysis. Panels A, B correspond to control reaction containing DDW for H3K56Ac and H3, respectively. Panels C, D and E correspond to the WT Sirt6 for H3K56Ac, H3 and Sirt6 detection, respectively. Panels F, G and H correspond to 6A4 mutant for H3K56Ac, H3 and Sirt6 detection, respectively. Panels I, J and K correspond to D1 mutant for H3K56Ac, H3 and Sirt6 detection, respectively.





**Fig. S12:** Original western blots for the IP analysis of D1 and 6A4 interaction with Hif1α and inhibition of gene expression relative to WT SIRT6 (**Fig. 6** main text). Extracts from HEK293 cells expressing Hif1α were IP with α-Myc antibodies conjugated to agarose beads followed by incubation with purified SIRT6 mutants or WT. Western blot analysis of the input of SIRT6, the IP SIRT6 and the levels of Hif1α are shown indicating higher interaction of D1 and 6A4 with Hif1α. The upper band in the middle of IP SIRT6 is due to antibody background from the beads used for the IP.


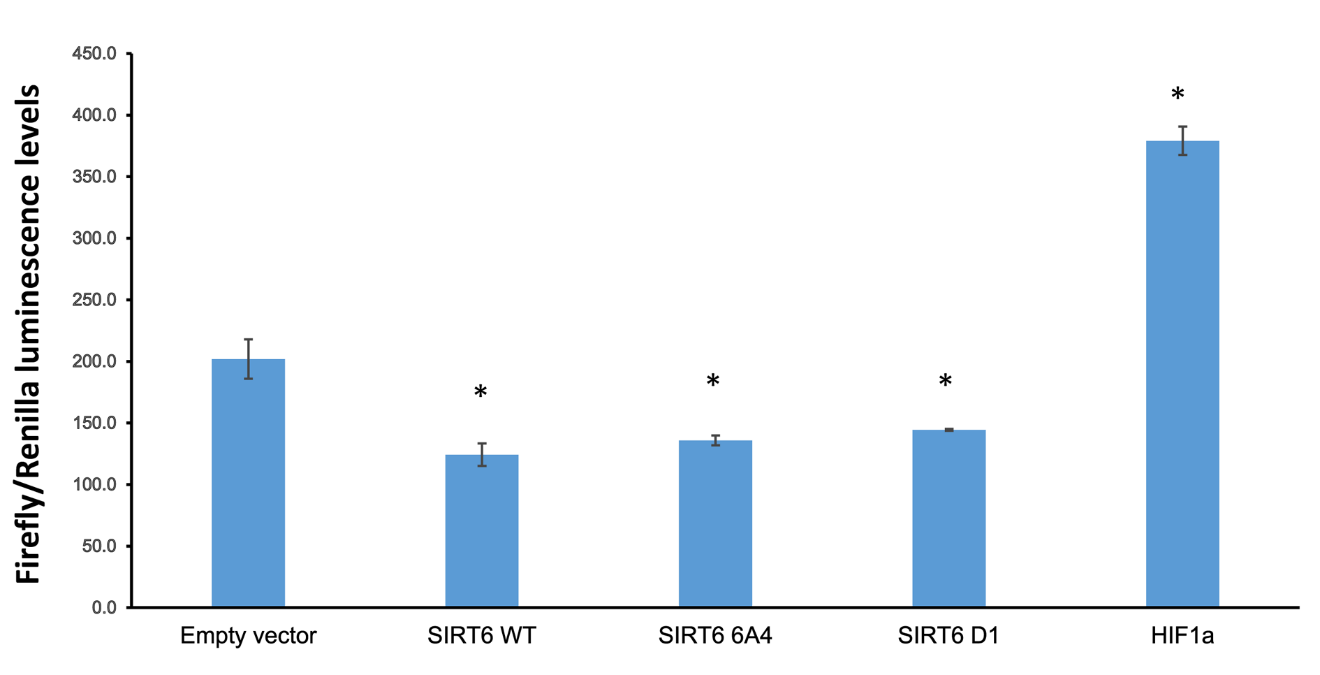


**Fig. S13**: SIRT6 mutants are able to repress Hif1α. A luciferase reporter gene under the regulation of three tandem copies of Hypoxia-Responsive Elements (HRE) was cotransfected with empty vector, SIRT6 WT, 6A4, D1 or Hif1α into SIRT6-KO 293T cell line (**Fig. S8**). Hif1α overexpression activates the expression of luciferase, confirming that these HRE elements indeed responds to Hif1α. In contrast, SIRT6 WT, 6A4 or D1 mutants showed reduced luciferase expression relative to empty vector highlighting their ability to repress Hif1α target genes even in the absence of deacetylation activity (**Fig. 4 and Fig. S9**). The data presented is the average of three independent technical repeats of each experiment while the error bars represent the standard deviation from the average. Statistically significant differences based on one way ANOVA analysis of the different SIRT6 variants relative to the empty vector levels are labeled with black star (p<0.01). No significant difference was observed between the WT and D1 or 6A4 variants,

| **Position** | **hSIRT6** | **Ancestor N6** | **Mutation** |
| --- | --- | --- | --- |
| **50** | H | M/L | H50M, H50L |
| **58** | A | S | A58S |
| **68** | H | V/S/G | H68V, H68S, H68G |
| **92** | T | S/N/Q | T92S, T92N |
| **93** | Q | P/L | Q93P, Q93L |
| **147** | Q | E | Q147E |
| **157** | M | Q/H | M157Q, M157H |
| **183** | D | P | D183P |
| **184** | T | D | T184D |
| **187** | D | F | D187F |
| **188** | W | F | W188F |
| **189** | E | G | E189G |
| **194** | D | P | D194P |
| **212** | T | V/C | T212V, T212C |
| **223** | G | A | G223A |
| **224** | N | S | N224S |
| **242** | Q | E | Q242E |
| **245** | K | P | K245P |
| **257** | Y | R | Y257R |
| **258** | V | C | V258C |

**Table S1**: Positions and mutations incorporated into SIRT6 ancestral library^a^.

^a^ Mutations were identified by comparing the hSIRT6 sequence to the ancestral N6 sequence focusing on residues that differ and are located up to 15Å from the catalytic histidine residue (H133).
